# Training Intensity Distribution of a 7-Day HIIT Shock Microcycle: Is Time in the “Red Zone” Crucial for Maximizing Endurance Performance? A Randomized Controlled Trial

**DOI:** 10.1186/s40798-024-00761-1

**Published:** 2024-09-05

**Authors:** Tilmann Strepp, Julia C. Blumkaitis, Mahdi Sareban, Thomas Leonhard Stöggl, Nils Haller

**Affiliations:** 1 Department of Sport and Exercise Science, University of Salzburg, Schlossallee 49, 5400 Hallein/Rif, Salzburg Austria; 2 University Institute of Sports Medicine, Prevention and Rehabilitation, University Hospital Salzburg, Paracelsus Medical University, Salzburg, Austria; 3 Red Bull Athlete Performance Center, Thalgau, Austria; 4 Department of Sport Medicine, Rehabilitation and Disease Prevention, Johannes Gutenberg University of Mainz, Mainz, Germany

**Keywords:** Time-in-zone, Running power, Running velocity, Heart rate, Block training, Polarized, Pyramidal, Cardiac remodeling, Athlete’s heart

## Abstract

**Background:**

Various studies have shown that the type of intensity measure affects training intensity distribution (TID) computation. These conclusions arise from studies presenting data from meso- and macrocycles, while microcycles, e.g., high-intensity interval training shock microcycles (HIIT-SM) have been neglected so far. Previous literature has suggested that the time spent in the high-intensity zone, i.e., zone 3 (Z3) or the “red zone”, during HIIT may be important to achieve improvements in endurance performance parameters. Therefore, this randomized controlled trial aimed to compare the TID based on running velocity (TID_V_), running power (TID_P_) and heart rate (TID_HR_) during a 7-day HIIT-SM. Twenty-nine endurance-trained participant were allocated to a HIIT-SM consisting of 10 HIIT sessions without (HSM, n = 9) or with (HSM + LIT, n = 9) additional low-intensity training or a control group (n = 11). Moreover, we explored relationships between time spent in Z3 determined by running velocity (Z3_V_), running power (Z3_P_), heart rate (Z3_HR_), oxygen uptake (${\text{Z}}{3_{\dot{\text{V}}\text{O}_2}}$) and changes in endurance performance.

**Results:**

Both intervention groups revealed a polarized pattern for TID_V_ (HSM: Z1: 38 ± 17, Z2: 16 ± 17, Z3: 46 ± 2%; HSM + LIT: Z1: 59 ± 18, Z2: 14 ± 18, Z3: 27 ± 2%) and TID_P_ (Z1: 50 ± 8, Z2: 14 ± 11, Z3: 36 ± 7%; Z1: 62 ± 15, Z2: 12 ± 16, Z3: 26 ± 2%), while TID_HR_ (Z1: 48 ± 13, Z2: 26 ± 11, Z3: 26 ± 7%; Z1: 65 ± 17, Z2: 22 ± 18, Z3: 13 ± 4%) showed a pyramidal pattern. Time in Z3_HR_ was significantly less compared to Z3_V_ and Z3_P_ in both intervention groups (all p < 0.01). There was a time x intensity measure interaction for time in Z3 across the 10 HIIT sessions for HSM + LIT (p < 0.001, _p_η^2^ = 0.30). Time in Z3_V_ and Z3_P_ within each single HIIT session remained stable over the training period for both intervention groups. Time in Z3_HR_ declined in HSM from the first (47%) to the last (28%) session, which was more pronounced in HSM + LIT (45% to 16%). A moderate dose–response relationship was found for time in Z3_V_ and changes in peak power output (r_s_ = 0.52, p = 0.028) as well as time trial performance (r_s_ = − 0.47, p = 0.049) with no such associations regarding time in Z3_P_, Z3_HR_, and ${\text{Z}}{3_{\dot{\text{V}}\text{O}_2}}$.

**Conclusion:**

The present study reveals that the type of intensity measure strongly affects TID computation during a HIIT-SM. As heart rate tends to underestimate the intensity during HIIT-SM, heart rate-based training decisions should be made cautiously. In addition, time in Z3_V_ was most closely associated with changes in endurance performance. Thus, for evaluating a HIIT-SM, we suggest integrating a comprehensive set of intensity measures.

*Trial Registration* Trial register: Clinicaltrials.gov, registration number: NCT05067426.

**Supplementary Information:**

The online version contains supplementary material available at 10.1186/s40798-024-00761-1.

## Key Points

- Using heart rate, running velocity and running power as measures of intensity leads to different patterns of training intensity distribution during a 7-day HIIT shock microcycle. A polarized pattern was observed for velocity and power, whereas a more pyramidal distribution was found for heart rate.
- Additional low-intensity training volume during a HIIT shock microcycle led to a more pronounced decline in time in zone 3 measured with heart rate compared to a shock microcycle with HIIT sessions, only.
- A moderate dose–response relationship was observed between time in zone 3 measured by velocity and changes in peak power output as well as time-trial performance. No such correlation was found between time in zone 3 measured by power, heart rate, oxygen uptake, and changes in endurance performance parameters.

**Supplementary Information:**

The online version contains supplementary material available at 10.1186/s40798-024-00761-1.

## Background

Training prescription typically relies on three key variables: frequency, volume, and intensity. The interaction of these variables can be used to quantify the training intensity distribution (TID) of an athlete [1]. To prescribe training intensity and calculate TID, various zones (Z) are typically determined by exercise testing. In this regard, the 3-zone model, i.e., Z1 (low intensity), Z2 (moderate intensity), and Z3 (high intensity) is most widely used in research [2, 3]. Up to 8 different TID patterns have been described in the literature [4, 5], with the most frequently observed patterns being pyramidal training (Z1 > Z2 > Z3), polarized training (Z1 > Z3 > Z2), and threshold training (Z2 > Z1 and Z3) [3, 4, 6]. There is evidence that TID impacts the training outcome [7–9]; however, it is heavily debated which TID is ultimately optimal for endurance athletes and their specific events [10, 11].

Training intensity can be assessed through measurements of external load (e.g., velocity, power output) [12], internal responses (e.g., heart rate (HR), oxygen uptake ($\dot{\text{V}}{{\text{O}}_2}$), blood lactate (La)), and subjective evaluations (e.g., rating of perceived exertion (RPE)) [13]. In cycling, for example, the combination of measuring power output and HR is well established [14, 15], while in running, intensity measures such as running velocity, HR or RPE are commonly used [16]. Methodological challenges like uphill or downhill running, day-to-day variability in internal responses, environmental factors, and overtraining may impact intensity measures and lead to possible over- or underestimations of the actual training intensity [17, 18]. In this respect, footpod devices that calculate running power may thus serve as reliable tools for measuring training intensity [19], yet their association with traditional intensity measures in longitudinal studies has not been sufficiently researched.

Various studies have shown that the type of intensity measure and the zone determination method substantially affects the TID computation [5, 20–22]. For instance, Bellinger et al. [21] collected 8 weeks of training data during the preparation phase from highly trained runners and found a polarized pattern for running velocity (80% Z1, 5% Z2, 15% Z3), a pyramidal pattern for HR (80% Z1, 17% Z2, 3% Z3), and a more uniform pattern for the RPE (40% Z1, 32% Z2, 28% Z3). Understanding these differences is important for athletes and coaches who rely on TID to make informed training decisions. Thus far, these conclusions primarily arise from studies that present data from meso- and macrocycles. However, microcycles play a crucial role in the process of endurance training as they allow to administer a specific stimulus in a short period of time, for example during preparation phase or when using a block periodization approach [23, 24].

High-intensity interval training shock microcycles (HIIT-SM), i.e., congested distribution of HIIT sessions in a short period of time, have been increasingly applied in endurance and team sports [25, 26]. With the aim to improve athlete performance in a time-efficient manner, studies demonstrated beneficial effects of HIIT-SM on maximal oxygen uptake (${\dot{\text{V}}}{\text{O}}_{{\text{2max}}}$) [26–28] or time-trial (TT) performance [29], while other findings have been less conclusive [24, 30, 31]. These discrepancies raise the question of what distinguishes an effective HIIT-SM from an ineffective one. Previous literature discussed that time spent in Z3 or the so-called “red zone”, i.e., ≥ 90% ${\dot{\text{V}}}{\text{O}}_{2\max}$/maximal heart rate (HR_max_) [32, 33], during HIIT may be important to achieve performance-related enhancements. It is suggested, to spend > 7 min (team sport athletes) or > 10 min (long-distance runners) [25, 32] near ${\dot{\text{V}}}{\text{O}}_{2\max}$ per session to elicit relevant changes in ${\dot{\text{V}}}{\text{O}}_{2\max}$. As the understanding of this dose–response relationship is still limited [30, 34, 35], these suggestions must be taken with caution. It is also unclear whether these conclusions are transferable to a HIIT-SM setting with or without additional low-intensity training (LIT). Reducing LIT during HIIT-SM has raised concerns about detrimental impact on performance development among practitioners, as LIT is seen as a crucial factor in an endurance athlete's routine [2]. However, it is unknown how additional LIT volume during HIIT-SM compared to a regular HIIT-SM affects the accumulation of time in Z3. In addition, most studies investigating the dose–response relationship between time in Z3 and increases in endurance performance rely on intensity measures, e.g., $\dot{\text{V}}{{\text{O}}_2}$ or HR, as they reflect the athlete’s responses that activate physiological mechanisms leading to training adaptation, while other measures such as running velocity or power have been neglected so far, although they might be more practical in application [32, 36].

Various factors such as training condition, sex, age or genetic predisposition may influence an athlete’s response to HIIT [32]. Another aspect that remains insufficiently explored is the impact of baseline cardiac geometry, as reflected by dimensions of cardiac chambers and cardiac wall thickness, at rest and endurance performance following a HIIT intervention. Indeed, no studies investigated whether baseline cardiac geometry variables predict HIIT-induced changes in endurance performance, specifically after HIIT-SM, or the time spent in Z3 in general. Considering that exercise-induced cardiac chamber enlargement enhances cardiac output during exercise primarily due to improved cardiac filling [37], and considering that maximal cardiac output is the major determinant of ${\dot{\text{V}}}{\text{O}}_{2\max}$ [38], small cardiac chambers at rest might indicate a greater reserve for adaption. Consequently, assessment of chamber geometry could enable more informed and personalized training recommendations.

Therefore, the current study compared the TID based on various intensity measures, i.e., running velocity (TID_V_), running power (TID_P_), HR (TID_HR_), and $\dot{\text{V}}{{\text{O}}_2}$, using a HIIT-SM consisting of 10 HIIT session in 7 days with or without 30 min of additional LIT after each session in endurance-trained athletes. In addition, we explored relationships between time spent in Z3 determined by running velocity (Z3_V_), running power (Z3_P_), HR (Z3_HR_), $\dot{\text{V}}{{\text{O}}_2}$ (${\text{Z}}{3_{\dot{\text{V}}\text{O}_2}}$) and changes in endurance performance. Finally, the influence of cardiac geometry on changes in endurance performance was examined. These findings contribute to identifying a possible dose–response relationship between training intensity and performance changes, thereby optimizing future HIIT-SM.

## Methods

This randomized controlled trial was registered (clinical trials identifier: NCT05067426). All procedures underwent approval from the local ethical board of the University of Salzburg (Approval Number: GZ 2/2021) and align with the principles of the Declaration of Helsinki. The comprehensive study protocol with all measures and detailed information is presented elsewhere [39]. The present manuscript was written in accordance with the CONSORT guidelines for randomized trials [40].

### Trial Design

**Fig. 1:**
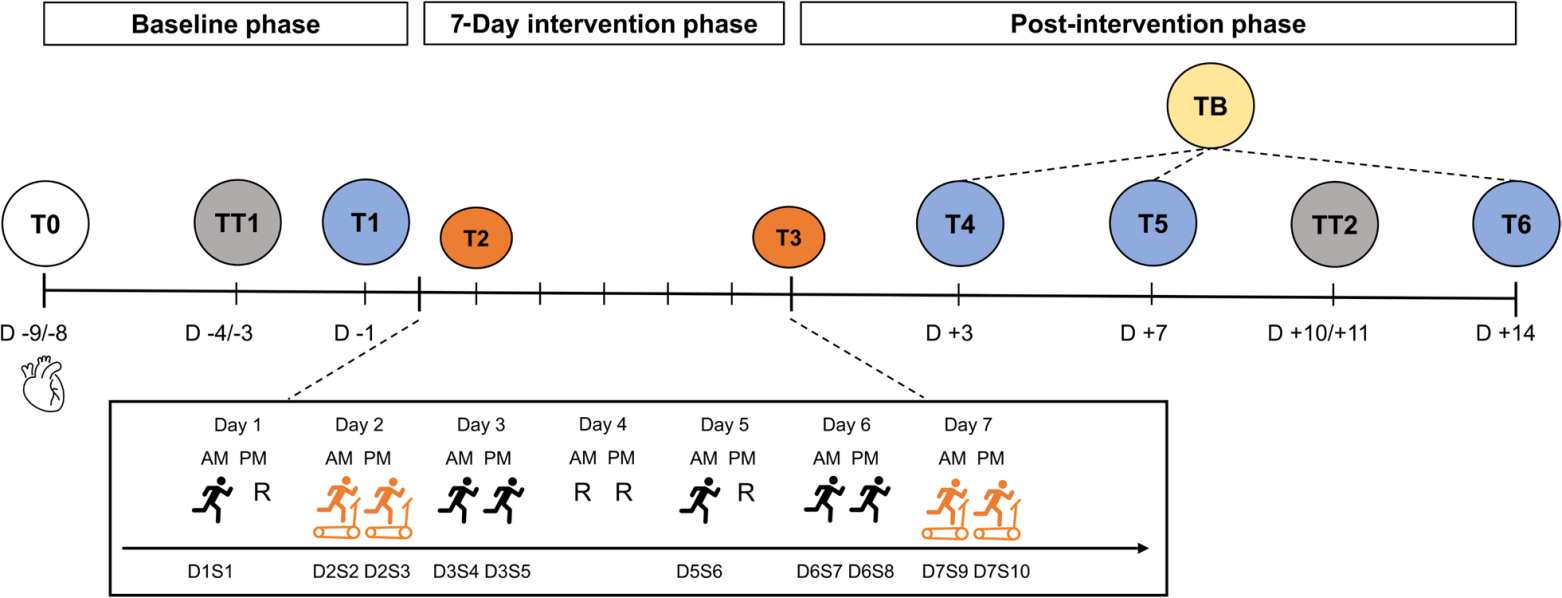
Outline of the study design with respective time points and the 7-day intervention phase. T0, familiarization and cardiac ultrasound; TT1 and TT2, 5-km time trial on an outdoor track; T1, T4, T5, T6, cardiopulmonary exercise testing on a treadmill; TB, best post measurement, i.e., maximum, or minimum value of each variable measured at post-tests T4, T5, T6, depending on what can be considered as an improvement in endurance performance; black runner, self-directed outdoor session; orange runner, supervised laboratory session; D, day; S, session; R, rest day

This is a parallel assignment intervention study in which participants were randomly assigned to one of two intervention groups or a control group (CG) in a 1:1:1 ratio. Total study duration was four weeks and included an initial baseline phase of 8 to 9 days, followed by a 7-day intervention phase and a 14-day post-intervention phase (Fig. 1).

At the initial time point (T0), all participants were familiarized with the study design, equipped with wearable devices, and underwent an ultrasound of the heart. Participants maintained their usual training routine throughout the baseline phase and completed a self-directed 5-km TT (TT_5km_) on a standardized track, either a 400 m running track or a flat course, at self-selected times, 3 to 4 days before their initial cardiopulmonary exercise testing on a treadmill at T1 (= TT1). After T1, participants were randomly allocated to one of 3 groups: (1) HIIT-SM with 10 HIIT sessions in 7 days (HSM), (2) HIIT-SM with 10 HIIT sessions and an additional 30 min of LIT after each session (HSM + LIT), or (3) a CG that maintained their regular training.

Four out of the 10 training sessions, i.e., 2 sessions on the second day (T2) and the final 2 sessions of the HIIT-SM on the last day (T3), were monitored in a laboratory setting. During the post-intervention phase, all participants underwent further cardiopulmonary exercise testing on a treadmill 3 days (T4), 7 days (T5), and 14 days (T6) after the intervention. Self-directed TT_5km_ was repeated on the same track 10 to 11 days (TT2) after the intervention (Fig. 1).

### Participants

Forty-three participants were recruited by announcements on social media, local sports clubs, and universities. Eligibility criteria included endurance-trained athletes, either runners or individuals who incorporate a significant amount of running (> 50 km per week) into their regular training. Participants had to be between 18 and 45 years old, with a ${\dot{\text{V}}}{\text{O}}_{2\max}$ of  ≥ 50 mL min^−1^ kg^−1^ (female) or  ≥ 55 mL min^−1^ kg^−1^ (male), or a TT_5km_ performance of  ≤ 20:00 min (female) or  ≤ 18:30 min (male). Supplement 1 provides detailed inclusion and exclusion criteria. All participants were informed about the aims and risks of the study and gave written consent before participating. Data collection took place from February 2021 to December 2022 in the exercise laboratory of the University of Salzburg.

### Intervention

**Fig. 2:**
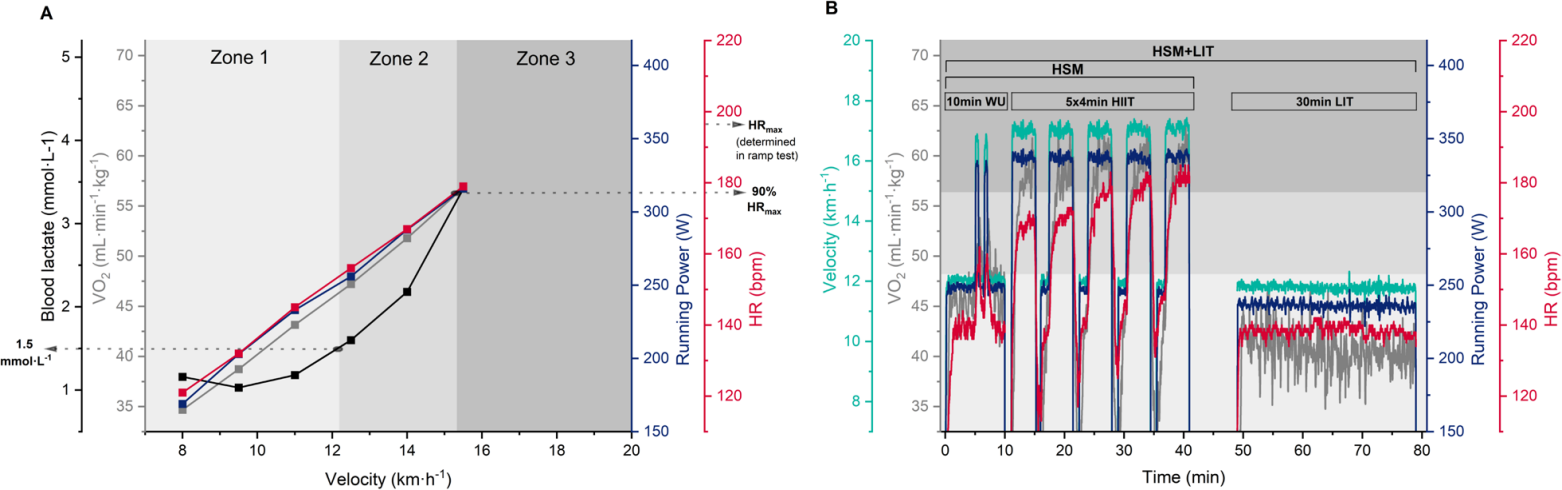
Exemplary data of a participant. **A** Incremental treadmill test at T1 with heart rate (HR), running power, oxygen uptake ($\dot{\text{V}}{{\text{O}}_2}$), velocity, and blood lactate values for each stage. Zones are separated by 1.5 mmol L^−1^ and 90% maximal heart rate. Maximal heart rate was determined via ramp test, performed after the incremental test. **B** Laboratory afternoon session on the second day (D2S3) including 10 min warm-up (WU), 5 × 4 min high-intensity interval training (HIIT), 30 min low-intensity training (LIT). HIIT shock microcycle (HSM) group only performed warm-up (WU) and HIIT, while the other group performed additional LIT after each session (HSM + LIT). Kinetics of each intensity measure and the respective time in zone is illustrated

HSM and HSM + LIT participants completed a total of 10 running HIIT sessions each consisting of 5 interval bouts of 4 min at an intensity associated with 90–95% HR_max_ measured at T1, interspersed by 2.5-min recovery periods (1-min passive and 1.5-min active recovery) (Fig. 2B) [41]. Sessions included a 10-min warm-up with two 30-s bouts at speeds associated with 90–95% HR_max_ measured at T1. Morning sessions took place between 6 and 10 AM. If 2 sessions were scheduled on the same day, the afternoon sessions were held from 3 to 7 PM, with a minimum of 5 h between sessions. Contrary to the HSM group, HSM + LIT participants performed 30 min of LIT after each HIIT session at an intensity that should not exceed the intensity associated with a La concentration of 1.5 mmol L^−1^ measured at T1. The HSM group was instructed not to perform a cool-down. All HIIT sessions were either self-monitored (6 out of 10) using individually programmed sessions on their watch with intensity guidance, i.e., HR targets, or were conducted under supervision on a treadmill in the laboratory at T2 and T3 (4 out of 10). The treadmill speed for the intervals was based on the velocity associated with 90–95% HR_max_ measured at T1. If necessary, the researchers increased the speed to achieve the HR targets, but only to a certain extent to ensure completion of the session. Participants in both groups were instructed not to perform any additional training sessions beyond the prescribed regimen. The CG continued with their regular training.

### Cardiac Examination

At T0, all participants underwent resting transthoracic cardiac ultrasound using a commercially available ultrasound system (Philips Epiq CVx, Philips Healthcare, Andover, MA, USA) with a 1.0–5.0 MHz xMatrix phased array transducer for both 2D and 3D imaging (Philips X5-1, Phillips Medical Systems, Andover, MA, USA) performed by a certified cardiologist to exclude relevant cardiac pathologies and to quantify variables of cardiac geometry. 2D- and 3D-derived diameters and volumetric measurements of cardiac chambers were performed according to current recommendations [42]. Volumetric 2D-measurements used the method of discs summation after manual tracing of endocardial boarders from apical 4-chamber view. Volumetric 3D-measurements were obtained by a software (HeartModel, Philips Healthcare, Andover, MA, USA) that involves an automated analysis which simultaneously detects left ventricular (LV) and left atrial (LA) endocardial boarders [43]. All echocardiographic measurements were indexed to body surface area calculated by the Mosteller formula [44]. Accepted cut-offs for normal heart geometry and eccentric hypertrophy, indicating exercise-induced cardiac remodeling, were used [42]. 

### Intensity Measures and Data Analysis

All participants received a Global Navigation Satellite System (GNSS) watch (Forerunner 935, Garmin, Kansas City, MO, USA), a HR chest strap (HRM Pro, Garmin, Kansas City, MO, USA), and a footpod (Wind v3, Stryd, Boulder, CO, USA) to measure running velocity, HR, and running power respectively during all outdoor and treadmill activities in the study period. The devices were paired and synchronized with the watch and data were sampled at 1 Hz for each intensity measure. Data were stored online (Garmin Connect, Garmin, Kansas City, MO, USA) and then imported into an open-source analysis software (GoldenCheetah v3.5) for further processing. All files were visually inspected for artefacts by an experienced sport scientist (TS). Training sessions with missing or incorrect data due to technical errors, e.g., connection issues or loss of signal, as well as uphill interval sessions were excluded from further analysis, which led to the exclusion of the respective participant (≥ 1 defective session = exclusion). Individual values for each zone and device were imputed into the software to calculate TID_V_, TID_P_, and TID_HR_.

For a total of 4 HIIT sessions (T2 AM/PM & T3 AM/PM), $\dot{\text{V}}{{\text{O}}_2}$ was sampled breath-by-breath using a Quark CPET (Cosmed, Rome, Italy). The system was volume-calibrated with a 3000 mL syringe and gas calibrated with a reference gas (16% O_2_, 5% CO_2_) before each session. Breath-by-breath data were processed using a custom code (Matlab v2021b, The MathWorks Inc., Natick, MA, USA). $\dot{\text{V}}{{\text{O}}_2}$ data were interpolated to calculate second-by-second values and filtered with a 10 s moving average to determine the time in zone based on $\dot{\text{V}}{{\text{O}}_2}$.

### Cardiopulmonary Exercise Testing

Cardiopulmonary exercise testing conducted at time points T1, T4, T5, and T6 (Fig. 1), were scheduled in the morning between 6 and 10 AM. Participants were instructed to abstain from vigorous exercise and alcohol for 24 h before each test. The testing protocol comprised a 2-phase approach on a treadmill (Saturn, HP Cosmos, Traunstein, Germany), starting with a sub-maximal incremental test (phase 1), followed by a ramp test to voluntary exhaustion (phase 2). Participants started the incremental test at 6.5 (females) and 8.0 km h^−1^ (males), with an incline of 0%. Speed increased by 1.5 km h^−1^ every 3 min. Between stages, 30-s pauses were used for capillary blood collection from the earlobe to assess La concentrations (Biosen S-Line, EKF Diagnostics GmbH, Magdeburg, Germany). The incremental test ended if any of the following criteria were met: (1) La increased by ≥ 1 mmol L^−1^ compared to the previous stage, (2) RPE > 17 on the 6–20 Borg-scale, or (3) the respiratory exchange ratio exceeded 1.0 for two consecutive stages. The ramp test speed was determined by the incremental test results (equal to the speed of the stage prior to the La increase) and remained constant with a steady increase in slope (1.5% per minute, starting at 0%) until voluntary exhaustion. A comprehensive description and illustration of the testing protocol can be found elsewhere [39].

The following endurance parameters were measured via exercise testing: (1) ${\dot{\text{V}}}{\text{O}}_{2\max}$ relative to body weight determined as the highest 10 breath rolling average; (2) Running peak power output (PPO) using the WOODWAY formula *(*1.065 + 0.0511 · %_max_ + 9.322 · 10^–4^ · %_max_^2^) · (v(TM) · 3.6) · BW/4, where the maximal treadmill grade (%_max_) was calculated by linear interpolation using the formula (%f – 1.5) + 1.5 · t/60, with %f being the treadmill grade of the last stage, v(TM) the velocity of the treadmill, and BW the individual body weight; (3) Lactate threshold (LT) defined as the velocity v where the delta value of La (v + 1.5) and La(v) reaches first time 1 mmol L^−1^ [39]; (4) Running economy, defined as $\dot{\text{V}}{{\text{O}}_2}$ in mL min^−1^ kg^−1^ at 11 km h^−1^ calculated as the average of the last 30 s of the stage [45].

### Intensity Zone Determination

The 3-zone model was based on a combination of La concentration and HR measures obtained during the 2-phase test at T1 (Fig. 2A). The La concentration of 1.5 mmol L^−1^ separated Z1 from Z2 [3]. 90% of HR_max_ separated Z2 from Z3 [46]. Corresponding values for each intensity measure, i.e., HR, running power, running velocity, $\dot{\text{V}}{{\text{O}}_2}$, were calculated with simple linear regression [47, 48]. The TID analysis for an exemplary session in the laboratory is illustrated in Fig. 2B.

### Sample Size and Randomization

A priori power analysis for repeated measures with a within-between interaction was conducted using G*Power (Version 3.1.9.7, Düsseldorf, Germany). Assuming a power of 0.8 and a medium effect size (Cohen’s f = 0.25) on ${\dot{\text{V}}}{\text{O}}_{2\max}$, in accordance with previous studies [49, 50], a minimum of 27 participants were required. To account for a potential dropout rate of 20–25%, an initial sample size of 36 participants was planned. However, due to a low dropout rate during the study, data collection was stopped after 35 participants were allocated. During the baseline phase and the exercise testing at T1, the participant, TS, and JB were blinded to the participant's individual allocation. Allocation information was disclosed immediately after the exercise testing at T1, provided all inclusion criteria were met, via a telephone call from an unbiased researcher (NH) using a concealed computer-generated allocation sequence (balanced, one block with a 1:1:1 ratio). Blinding was not applicable for participants or researchers after allocation.

### Statistics

Results are presented as mean ± standard deviation (SD). In addition, the standard error of the mean (SEM) is given for the respected values shown in the figures. A repeated measures analysis of variance was performed to analyze differences between (1) changes in endurance performance parameters from T1 and the best (TB) out of three post-tests (T4, T5, T6) for each parameter (time) and group (HSM, HSM + LIT, CG); (2) zone (Z1, Z2, Z3), intensity measure (HR, velocity, power) and group (HSM, HSM + LIT, CG); (3) time spend in Z3 for all HIIT sessions (time) and intensity (HR, velocity, power) for both HSM and HSM + LIT separately; (4) time spend in Z3 for all laboratory HIIT sessions (time) and intensity (HR, velocity, power, $\dot{\text{V}}{{\text{O}}_2}$) with pooled intervention groups.

Whenever sphericity was not given (Mauchly Test, p < 0.05), Greenhouse–Geisser correction was applied. Alpha level of significance was set to < 0.05. Effect sizes by means of partial eta squared (_p_η^2^) are provided. In case of a significant main and/or interaction effect, further post-hoc analysis was performed. All tests were adjusted by Bonferroni correction. For comparison (1) data from all training sessions (including warm-up, HIIT and LIT) during the intervention phase were used, while for comparison (2) and (3) only HIIT sessions were analyzed. It should also be noted that running velocity and running power data was not applicable to CG, as they were allowed to continue with their regular training, which included other sports, e.g., cycling or swimming, besides running.

To estimate dose–response relationships, change scores (Δ %) from baseline performance parameters (T1) and TB for each parameter were calculated (Fig. 1). This was based on the assumption that participants would individually demonstrate time-delayed peaks in various performance parameters [51]. Relationships between baseline performance, change scores in performance, time in Z3 for each intensity measure, and cardiac geometry variables for each group separately and with pooled intervention groups were analyzed using Spearman’s rho (r_s_) [52]. The Statistical Package for the Social Science (SPSS, v27.0, IBM, Chicago, IL, USA), R-Studio (v2023.03.0 Build 386) and OriginPro (v2023, OriginLab Corporation, Northampton, MA, USA) were used for statistical analysis and the creation of figures.

## Results

Out of a total of 43 recruited participants during February 2021 and December 2022, 8 participants did not meet the inclusion criteria. Of the 35 assigned participants, 29 (23 male and 6 female) were eligible for data analysis after excluding 2 dropouts and 4 participants due to insufficient data quality. Supplement 2 provides a detailed participant flow chart. Anthropometric and cardiac geometry data as well as endurance performance data are presented in Tables 1 and 2. Twenty-six participants (89.7%) had normal cardiac geometry. Three participants (10.3%) showed eccentric hypertrophy. None of the participants showed functional abnormalities of the heart. HSM completed on average 49.9 ± 0.3 (99.8%) and HSM + LIT 49.3 ± 1.3 (98.7%) intervals. Average training time for the intervention phase was 7.4 ± 0.1 h for HSM, 12.3 ± 0.1 h for HSM + LIT, and 8.4 ± 1.1 h for CG. Average running distance was 90.9 ± 8.6 km for HSM, 144.7 ± 17.3 km for HSM + LIT, and 44.6 ± 18.8 km for CG.

**Table 1:** Anthropometric and cardiac geometry data (mean ± SD)

	Anthropometric data			Cardiac geometry										
				2D							3D*			
	Age (years)	Height (cm)	Weight (kg)	LV internal EDD (mm m^2^)	LV EDV (mL m^2^)	LV ESV (mL m^2^)	LV mass (g m^2^)	LA ESV (mL m^2^)	RV basal diameter (mm m^2^)	RA ESV (mL m^2^)	LV EDV (mL m^2^)	LV ESV (mL m^2^)	LV mass (g m^2^)	LA ESV (mL m^2^)
Participants (n = 29)	30 ± 6	177 ± 8	69.2 ± 8.7	28.8 ± 2.3	55.3 ± 10.2	22.8 ± 6.9	89.3 ± 15.0	27.1 ± 8.2	22.0 ± 2.4	26.4 ± 7.8	99.3 ± 10.6	40.3 ± 7.6	81.8 ± 9.7	35.3 ± 7.0
HSM (n = 9)	30 ± 5	173 ± 9	63.1 ± 5.5	28.8 ± 2.7	48.7 ± 5.3	19.2 ± 4.4	76.4 ± 10.7	22.6 ± 7.0	22.1 ± 2.2	25.4 ± 6.4	93.0 ± 6.8	38.6 ± 6.1	77.3 ± 7.6	31.9 ± 7.6
Female (n = 4)	30 ± 8	167 ± 3	58.4 ± 3.4	29.7 ± 1.7	46.1 ± 3.6	19.2 ± 1.5	73.2 ± 9.7	22.3 ± 4.8	21.7 ± 2.0	24.2 ± 5.5	89.2 ± 6.6	34.7 ± 2.2	77.2 ± 9.7	29.3 ± 5.9
Male (n = 5)	30 ± 2	178 ± 8	66.8 ± 3.5	28.0 ± 3.3	50.8 ± 5.6	19.2 ± 6.2	79.1 ± 11.7	22.8 ± 9.1	22.4 ± 2.6	26.4 ± 7.5	95.3 ± 6.5	40.9 ± 6.7	77.3 ± 7.4	33.4 ± 8.7
HSM + LIT (n = 9)	29 ± 7	179 ± 8	72.1 ± 7.4	28.6 ± 1.9	61.2 ± 13.1	26.1 ± 8.2	95.0 ± 15.7	31.4 ± 9.1	22.1 ± 2.8	24.5 ± 9.1	103.0 ± 10.9	42.7 ± 7.1	82.8 ± 6.8	39.1 ± 5.3
Male (n = 9)	29 ± 7	179 ± 8	72.1 ± 7.4	28.6 ± 1.9	61.2 ± 13.1	26.1 ± 8.2	95.0 ± 15.7	31.4 ± 9.1	22.1 ± 2.8	24.5 ± 9.1	103.0 ± 10.9	42.7 ± 7.1	82.8 ± 6.8	39.1 ± 5.3
CG (n = 11)	30 ± 7	180 ± 8	71.9 ± 9.8	29.2 ± 2.4	56.0 ± 8.0	22.9 ± 6.6	95.0 ± 11.4	27.1 ± 6.9	21.9 ± 2.3	28.8 ± 7.7	101.0 ± 11.4	39.5 ± 9.0	84.6 ± 12.7	34.7 ± 6.9
Female (n = 2)	25 ± 1	171 ± 9	60.4 ± 18.5	32.1 ± 3.7	54.2 ± 11.2	17.8 ± 8.9	95.7 ± 22.1	30.2 ± 15.9	22.5 ± 1.0	26.7 ± 5.4	96.8 ± 12.7	36.2 ± 6.0	74.8 ± 13.3	34.5 ± 6.4
Male (n = 9)	32 ± 8	181 ± 6	74.4 ± 6.1	28.5 ± 1.7	56.4 ± 8.0	24.1 ± 6.0	94.9 ± 10.1	26.5 ± 5.0	21.7 ± 2.5	29.3 ± 8.3	102.0 ± 11.7	40.3 ± 9.8	87.0 ± 12.1	34.8 ± 7.4

HSM, high-intensity shock microcycle; HSM + LIT, high-intensity shock microcycle with additional low-intensity training; CG, control group; 2D, two-dimensional; 3D, three-dimensional; LV internal EDD, left ventricular internal end-diastolic diameter; LV EDV, left ventricular end-diastolic volume; LV ESV, left ventricular end-systolic volume; LV mass, left ventricular mass; LA ESV, left atrial end-systolic volume; RV, right ventricular; RA ESV, right atrial end-systolic volume. All cardiac measurements were indexed to body surface area

^*^ Image quality of two participants did not allow software-based 3D-analyses

**Table 2:** Baseline and change in endurance performance parameters (mean ± SD [range])

HSM (n = 9)			HSM + LIT (n = 9)			CG (n = 11)			ANOVA (p/_p_η2)	
T1	TB	Δ (%)	T1	TB	Δ (%)	T1	TB	Δ (%)	Time	Time x Group
${\dot{\text{V}}}{\text{O}}_{2\max}$ (mL min^−1^ kg^−1^)										
59.2 ± 6.5	62.9 ± 5.4*	+ 6.5 ± 7.0	61.5 ± 3.8	64.7 ± 6.4*	+ 5.2 ± 6.2	59.4 ± 2.5	59.8 ± 3.2	+ 0.7 ± 3.8	** < .001/.37**	.076/.18
[51.2–70.0]	[55.2–71.3]	[− 0.7 to 20.4]	[55.5–66.6]	[56.8–76.5]	[− 2.0 to 14.9]	[54.8–62.4]	[55.9–65.0]	[− 6.4 to 4.9]		
PPO (W kg^−1^)										
5.01 ± 0.67	5.26 ± 0.72*	+ 4.9 ± 3.2	5.27 ± 0.33	5.46 ± 0.23*	+ 3.7 ± 2.7	4.99 ± 0.36	5.12 ± 0.46*	+ 2.5 ± 3.2	** < .001/.63**	.259/.10
[4.04–6.31]	[4.39–6.66]	[− 0.09 to 10.1]	[4.69–5.70]	[4.99–5.75]	[− 0.83 to 6.41]	[4.44–5.60]	[4.30–5.78]	[− 3.18 to 9.42]		
LT (km h^−1^)										
12.3 ± 2.0	12.9 ± 1.6*	+ 5.3 ± 4.2	12.7 ± 1.4	13.2 ± 1.2*	+ 4.2 ± 3.7	12.5 ± 1.0	12.7 ± 1.0	+ 1.6 ± 2.6	** < .001/.59**	.071/.18
[9.7.1–15.6]	[10.6–15.6]	[0.0–12.1]	[9.9–14.2]	[11.0–14.4]	[− 0.8 to 11.1]	[11.0–13.6]	[11.2–14.4]	[− 3.0 to 5.9]		
Economy (mL min^−1^ kg^−1^)										
38.6 ± 5.7	36.0 ± 2.7	− 5.8 ± 8.5	42.1 ± 2.9	39.5 ± 2.5	− 6.1 ± 2.0	39.3 ± 3.0	37.1 ± 2.3	− 5.2 ± 5.8	** < .001/.51**	.895/.01
[30.8–46.2]	[32.4–40.2]	[− 14.7 to 8.1]	[37.9–45.3]	[36.0–43.0]	[− 9.4 to − 1.9]	[34.7–44.0]	[34.7–41.5]	[− 15.3 to 4.7]		

TT1	TT2	Δ (%)	TT1	TT2	Δ (%)	TT1	TT2	Δ (%)		
TT_5km_ (mm:ss)										
19:20 ± 2:31	19:02 ± 2:18*	− 1.5 ± 1.6	18:56 ± 1:23	18:27 ± 1:11*	− 2.3 ± 2.7	19:04 ± 1:24	19:07 ± 1:38	+ 0.1 ± 2.1	**.007/.25**	.053/.20
[15:05–23:04]	[14:57–22:15]	[− 4.6 to 0.3]	[17:12–21:56]	[17:20–20:39]	[− 5.9 to 2.8]	[16:48–21:04]	[16:29–22:10]	[− 1.9 to 5.2]		

HSM, high-intensity shock microcycle; HSM + LIT, high-intensity shock microcycle with additional low-intensity training; CG, control group; ${\dot{\text{V}}}{\text{O}}_{2\max}$, maximal oxygen uptake; T1, baseline timepoint; TB, best out of 3 posttests; Δ, changes from pre to best posttest; PPO, peak power output; LT, lactate threshold; TT, 5 km time trial; TT1, pre 5 km time trial; TT2, post 5 km time trial

*Significant (p < 0.05) different to T1; bold font, significant main effect

### Training Intensity Distribution

**Fig. 3:**
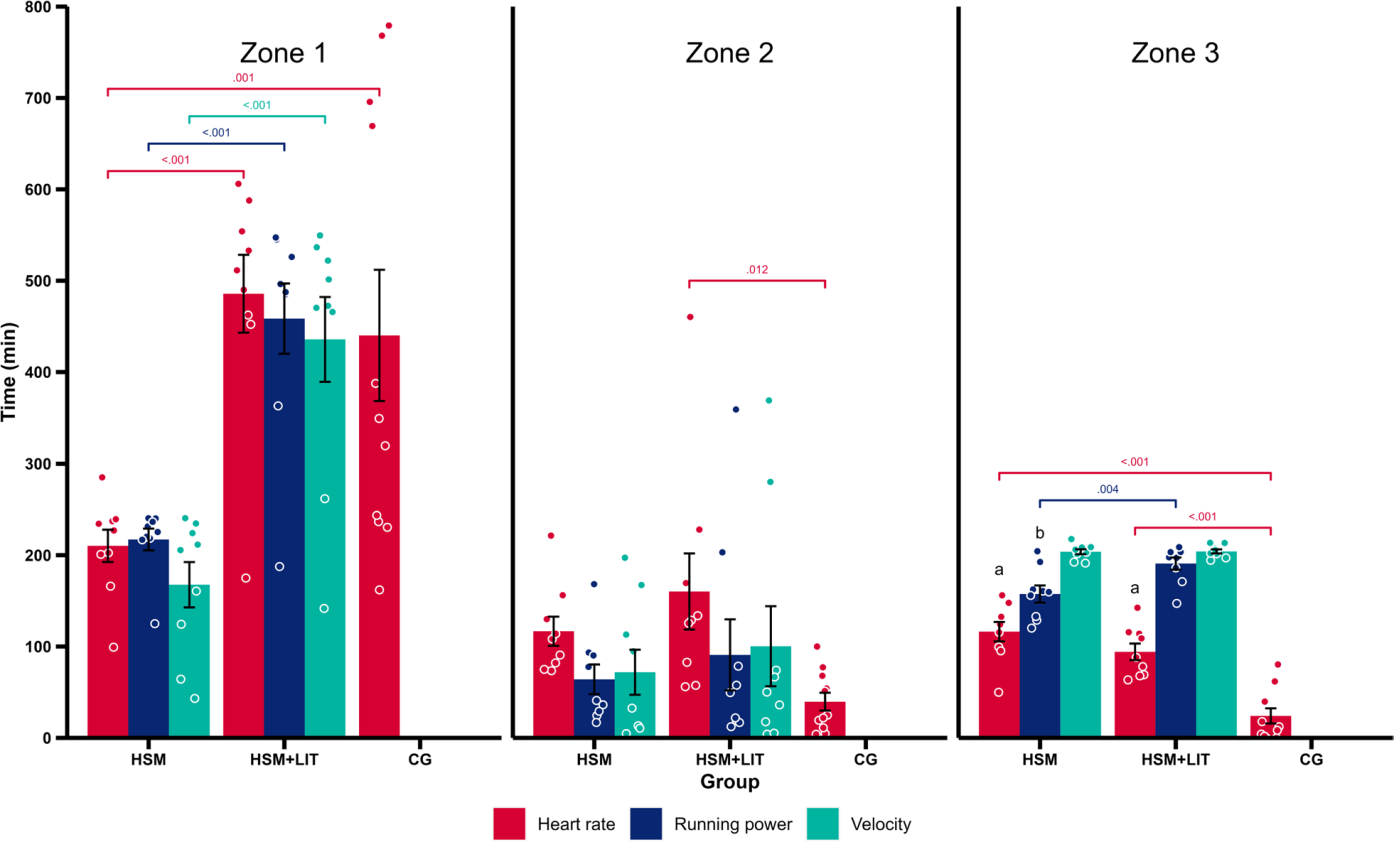
Training intensity distribution of the 7-day intervention phase for each group and intensity measure. Mean (bars), individual data (points), and standard error of the mean (whiskers). Zones were identified from exercise testing with corresponding values for 1.5 mmol L^−1^ blood lactate and 90% maximal heart rate. Pairwise comparisons between groups for each intensity measure within the 3 zones are displayed in the corresponding color. Comparison within each group and zone are displayed with characters. a = heart rate is different (p < 0.05) from running power and velocity within the respective group and zone; b = running power is different (p < 0.05) from velocity within the respective group and zone. HSM, high-intensity interval training shock microcycle; HSM + LIT, high-intensity interval training shock microcycle with additional low-intensity training; CG, control group

The mean upper limits for Z1 for HR, running power, running velocity, and $\dot{\text{V}}{{\text{O}}_2}$ were 154 ± 9 bpm, 253 ± 52 W, 11.8 ± 1.7 km h^−1^, and 42.1 ± 5.0 mL min^−1^ kg^−1^, whereas the mean upper limits for Z2 were 171 ± 8 bpm, 299 ± 46 W, 14.0 ± 1.3 km h^−1^, and 49.0 ± 4.4 mL min^−1^ kg^−1^. TID_V_, TID_P_, and TID_HR_ are presented in Fig. 3 including pairwise comparisons. There was a large effect for zone (p < 0.001, _p_η^2^ = 0.64), group (p < 0.001, _p_η^2^ = 0.72), zone × group (p < 0.001, _p_η^2^ = 0.36), and a medium effect for zone × intensity measure (p = 0.037, _p_η^2^ = 0.10). For both intervention groups a polarized pattern for TID_V_ (HSM: Z1: 38 ± 17, Z2: 16 ± 17, Z3: 46 ± 2%; HSM + LIT: Z1: 59 ± 18, Z2: 14 ± 18, Z3: 27 ± 2%) and TID_P_ (Z1: 50 ± 8, Z2: 14 ± 11, Z3: 36 ± 7%; Z1: 62 ± 15, Z2: 12 ± 16, Z3: 26 ± 2%) was found, whereas TID_HR_ was pyramidal (Z1: 48 ± 13, Z2: 26 ± 11, Z3: 26 ± 7%; Z1: 65 ± 17, Z2: 22 ± 18, Z3: 13 ± 4%). TID_HR_ (Z1: 84 ± 16, Z2: 10 ± 10, Z3: 6 ± 7%) for CG, who continued with their regular training, also showed a pyramidal pattern. ${\text{TI}}{{\text{D}}_{\dot{\text{V}}{{\text{O}}_2}}}$ measured during the four laboratory HIIT sessions excluding warm-up and LIT showed a polarized pattern for HSM (Z1: 33 ± 7, Z2: 20 ± 12, Z3: 46 ± 14%) and a pyramidal pattern for HSM + LIT (Z1: 39 ± 10, Z2: 31 ± 20, Z3: 30 ± 18%).

**Fig. 4:**
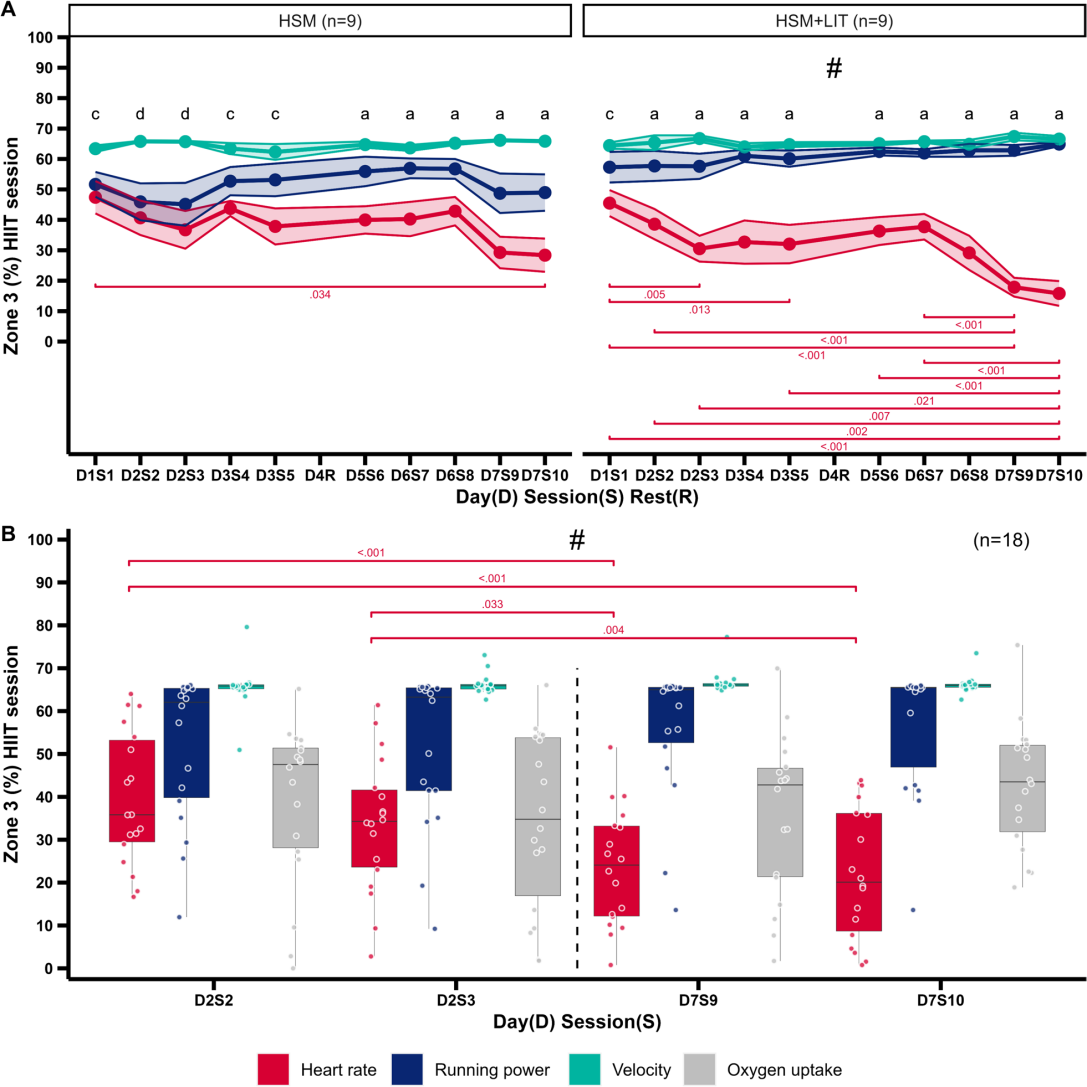
**A** Line plot with mean (dots) and standard error of the mean (band) presenting the time in zone 3 (Z3) over the 10 HIIT sessions for 3 intensity measures (heart rate (HR), running power (P), velocity (V)) and both groups as session percentages. # = significant interaction effect (time × intensity). Pairwise comparisons between sessions are presented including exact p-values. Pairwise comparisons between intensity measures for each session are marked with lower characters (p < 0.05): a = HR different to P and V, b = P different to V, c = HR different to V, d = V different to P and HR. **B** Boxplot of Z3 with individual data (circles) over the four laboratory HIIT sessions for HR, P, V, and oxygen uptake with pooled intervention groups

Figure 4A presents time in Z3_V_, Z3_P_, and Z3_HR_ during the 10 HIIT sessions for both intervention groups. HSM showed a large effect for intensity measure (p < 0.001, _p_η^2^ = 0.64), but neither a time (p = 0.132, _p_η^2^ = 0.07) nor a time x intensity measure (p = 0.149, _p_η^2^ = 0.11) effect. For HSM + LIT, no time effect (p = 0.080, _p_η^2^ = 0.08), but large effects for intensity measure (p < 0.001, _p_η^2^ = 0.85) and time x intensity measure were found (p < 0.001, _p_η^2^ = 0.30). Time in Z3_HR_ was less compared with their counter parts for most sessions and showed a more pronounced decline over the 10 sessions for HSM + LIT compared to HSM. Mean time in Z3_HR_ percentages of the 10 HIIT sessions were ranging between 28 and 47% for HSM and 16 and 45% for HSM + LIT, while time in Z3_P_ percentages were ranging from 45 to 57% and 57 to 63%. Time in Z3_V_ percentages over the intervention phase for both groups were ranging between 62 and 67% (Fig. 4A).

Figure 4B presents time in Z3_V_, Z3_P_, Z3_HR_, ${\text{Z}}{3_{\dot{\text{V}}\text{O}_2}}$ as a percentage during the 4 HIIT sessions in the laboratory. Average interval speeds were 14.6 ± 1.3, 14.6 ± 1.4, 14.8 ± 1.4, 14.9 ± 1.4 km h^−1^ for the 4 sessions, respectively. Analysis revealed no time (p = 0.156, _p_η^2^ = 0.03), but a large effect of intensity measure (p < 0.001, _p_η^2^ = 0.59) as well as a time × intensity measure (p < 0.001, _p_η^2^ = 0.20) effect for the pooled sample. There was a decline in time in Z3_HR_ with an average of 40 ± 16, 34 ± 16, 24 ± 14, and 22 ± 15% for the 4 sessions, respectively. Time in Z3_V_, Z3_P_, and ${\text{Z}}{3_{\dot{\text{V}}\text{O}_2}}$ showed no significant pairwise comparisons over time. Athletes spent on average 11 ± 6, 10 ± 6, 11 ± 6, and 13 ± 4 min in ${\text{Z}}{3_{\dot{\text{V}}\text{O}_2}}$ for the 4 sessions, respectively.

### Correlation Analysis

**Fig. 5:**
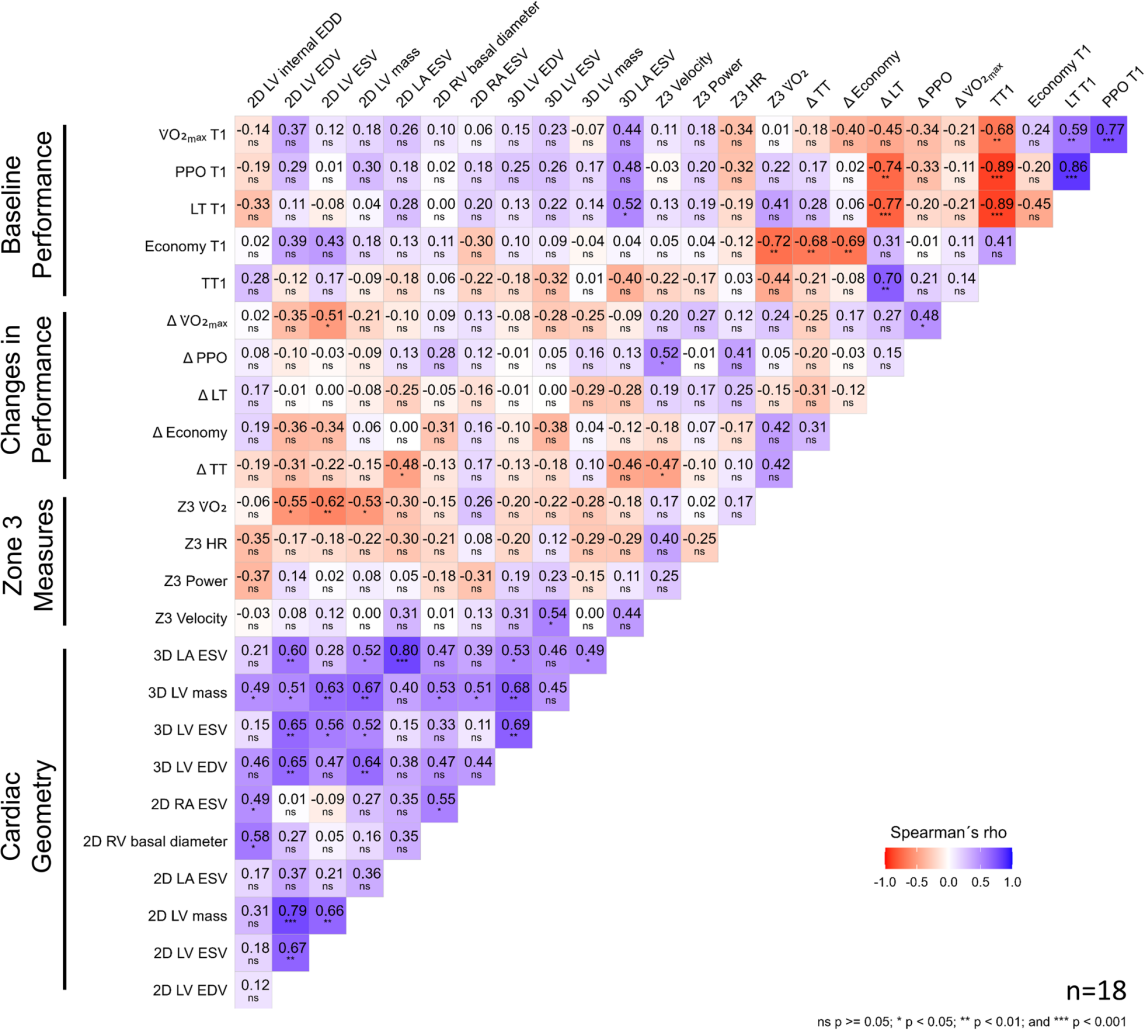
Correlation matrix (spearman rank) with baseline values and changes in performance measures, total time in zone 3 in minutes (Z3) determined with different intensity measures, and cardiac geometry variables with pooled intervention groups. T1, initial cardiopulmonary exercise testing; ${\dot{\text{V}}}{\text{O}}_{2\max}$, maximal oxygen uptake; Δ, changes from pre to best posttest; PPO, peak power output; LT, lactate threshold; TT, 5 km time trial; 2D, two-dimensional; 3D, three-dimensional; LV internal EDD, left ventricular internal end-diastolic diameter; LV EDV, left ventricular end-diastolic volume; LV ESV, left ventricular end-systolic volume; LV mass, left ventricular mass; LA ESV, left atrial end-systolic volume; RV, right ventricular; RA ESV, right atrial end-systolic volume. All cardiac measurements were indexed to body surface area. Image quality of two participants did not allow software-based 3D-analyses

The following correlation matrix (Fig. 5) shows pairwise correlations between selected variables. There were moderate to strong negative and positive correlations within baseline performance measures, except for running economy. Some baseline performance measures also showed moderate to strong correlations with Δ TT, Δ Economy, and Δ LT, while none were found with Δ ${\dot{\text{V}}}{\text{O}}_{2\max}$ and Δ PPO. Participants with a lower running economy, i.e., higher relative $\dot{\text{V}}{{\text{O}}_2}$ at 11 km h^−1^, at T1 showed less time in ${\text{Z}}{3_{\dot{\text{V}}\text{O}_2}}$ (r_s_ = − 0.72, p = 0.003). The analysis revealed no significant relationship between time in Z3_V_, Z3_P_, Z3_HR_ and changes in performance measures, except for Z3_V_ with Δ TT (r_s_ = − 0.47, p = 0.049) and Δ PPO (r_s_ = 0.52, p = 0.028).

Several correlations were observed among cardiac geometry variables. For instance, there was a strong correlation between LV mass and LV end-diastolic volume (EDV) (r_s_ = 0.79, p < 0.001) and between 2D- with 3D measurements, which is unsurprising given that they derive from similar data sources or measure the same cardiac structure, respectively. Further consistent (2D and 3D results) moderate relationships were found between LA end-systolic volume (ESV) and Δ TT (2D: r_s_ = − 0.48, p = 0.045, 3D: r_s_ = − 0.46, p = 0.066). Supplement 3 provides further detailed correlation analyses for each group separately between the variables.

## Discussion

A polarized pattern was revealed for TID_V_ and TID_P_ for both intervention groups, whereas TID_HR_ showed a pyramidal pattern. Time in Z3_HR_ was significantly less compared to time in Z3_V_ and Z3_P_ for HSM and HSM + LIT. In addition, time in Z3_V_ and Z3_P_ remained relatively consistent across the 10 HIIT sessions, whereas Z3_HR_ showed a decline in both groups that was more pronounced in HSM + LIT. More importantly, time in Z3_V_ demonstrated a moderate dose–response relationship with Δ PPO and Δ TT, indicating that athletes who accumulate more time in Z3_V_ tend to improve their PPO and TT performance to a greater extent. In contrast, there were no relationships between time in Z3_P_, Z3_HR_, ${\text{Z}}{3_{\dot{\text{V}}\text{O}_2}}$ and changes in endurance performance parameters. Finally, there were no relationships between cardiac geometry and baseline endurance performance parameters as well as change scores with the exception of LA ESV and Δ TT.

### TID of a HIIT Shock Microcycle

For the first time, we present data on the TID of a 7-day HIIT-SM with or without additional LIT based on various intensity measures. As expected, HSM + LIT participants achieved more time in Z1 for all three intensity measures compared to HSM, while there was no difference between HSM + LIT and CG concerning time in Z1_HR_ (Fig. 3). However, both HSM and HSM + LIT showed a polarized pattern for TID_V_ and TID_P_, whereas TID_HR_ was rather pyramidal. At the same time, TID_HR_ for CG, who continued with their regular training, also showed a pyramidal pattern. These differences in TID between intensity measures arise from both intervention groups achieving less time in Z3_HR_ compared to Z3_V_ and Z3_P_. Subsequently, this has led to an increase in time spent in Z2_HR_; a shift that can mainly be attributed to slower HR kinetics during the start of each interval compared with running velocity and power kinetics (Fig. 2B). Although comparisons between studies are difficult because most trials report training data of mesocycles (8–10 weeks), similar TID patterns have been observed in cyclists [20], middle-distance runners [21] or kayakers [22]. There is consensus that quantifying TID with different intensity measures will most likely lead to different distributions [3], which needs to be considered when making informed training decisions.

By analyzing Z3 data across all HIIT sessions (Fig. 4A), it can be concluded that the effect of delayed HR kinetics on time in Z3_HR_ was enhanced with an increasing number of HIIT sessions. In contrast, time in Z3_V_ and Z3_P_ remained relatively constant, indicating that most athletes still managed to achieve Z3 work rates based on velocity and power, but that these were underestimated in Z3_HR_. Interestingly, the decline in time in Z3_HR_ was more pronounced in HSM + LIT compared to HSM, already evident after session 3 (31 ± 4%, compared to session 1 45 ± 4%, p = 0.005). However, from the first to the last session, time in Z3_HR_ was decreased in both, HSM (47 ± 5% to 28 ± 5%, p = 0.034) and HSM + LIT (45 ± 4% to 16 ± 4%, p < 0.001). Differences in HR responses between the groups could be attributed to the additional training load of 300 min of LIT in the HSM + LIT group, increasing the strain in the athlete’s body. Of note, both groups showed a slight upward trend for Z3_HR_ following the rest day for 2 to 3 sessions before declining again, suggesting a potential recovery effect. In addition, athletes reported that towards the end of the HIIT-SM they had reached a limit where they could not run faster to meet the HR targets without risking the completion of the entire session. The decrease in time in Z3_HR_ towards the end is also reflected in a reduced HR_max_ (4–6 bpm) 3 days after the intervention, which has been presented previously [51]. The observation of a decrease in HR_max_ after intense training aligns with previous studies [26, 53]. Such observations are attributed to the accumulated fatigue and disruptions in the autonomic nervous system, particularly a decline in sympathetic nervous system activity [54, 55]. It can be argued, whether this reaction is desirable and necessary to induce performance improvements [56]. Interestingly, athletes showed notable discrepancies in time spent in Z3_HR_, with a 106 min (HSM) and a 78 min difference (HSM + LIT) between those with the longest and shortest durations (Fig. 3). Further research is needed to explore individual factors that may explain why some individuals experience greater declines than others. It is crucial for athletes and coaches to consider these mechanisms when interpreting the TID_HR_ of a HIIT-SM [57]. Our results also indicate that additional LIT, i.e., long cool-downs, are counterproductive if the goal is to maximize time in Z3_HR_ during HIIT-SM.

Most studies investigating running power data derived by Stryd featured a laboratory setting [19, 47, 58]. Here we present a longitudinal training dataset of running power in a “real-world” setting, with outdoor and indoor training sessions. In line with previous research [47, 59, 60], our results mostly support the linear relationship between running power and running velocity in this applied setting. There were no differences in time in zone between power and velocity within each group and zone, except for Z3 in HSM (Fig. 3). Figure 4A, B outline that especially the sessions in the laboratory resulted in differences between measures. This finding may be due to fixed interval speeds on the treadmill, were some cases partly missed the Z3_P_ threshold, but at the same time accumulated time in Z3_V._ More importantly, participants were instructed to run their outdoor sessions on a flat course, what benefits the linear power-velocity relationship. In contrast, varying gradients and speeds would likely have resulted in different patterns of TID_P_ and TID_V_ which warrants further investigation in future studies.

Analysis of ${\text{Z}}{3_{\dot{\text{V}}\text{O}_2}}$, Z3_P_, and Z3_V_ data from double session days in the laboratory with pooled intervention groups revealed no change over time, whereas Z3_HR_ decreased between T2 and T3 (Fig. 4B). Each intensity measure demonstrated its own characteristics in terms of time in Z3 and response to accumulated load during HIIT-SM. Due to faster kinetics during intervals (also see Fig. 2B) and no response to accumulated load and fatigue during the HIIT-SM, both running velocity and power achieved the most time in Z3 across all laboratory sessions. In contrast, the internal measures HR and $\dot{\text{V}}{{\text{O}}_2}$ achieved on average less time in Z3 with $\dot{\text{V}}{{\text{O}}_2}$ being consistent across the analyzed sessions, indicating comparable amount of metabolic work for the intervals. Contrary, HR was responsive to the accumulated load and showed a decline in Z3_HR_. It is important to consider these factors when interpreting TID based on different intensity measures, as they primarily influence the outcome. In addition, there was a high variability between athletes in achieving time in ${\text{Z}}{3_{\dot{\text{V}}\text{O}_2}}$ (time above $\dot{\text{V}}{{\text{O}}_2}$ at 90% HR_max_ measured at T1), although associated interval speeds were respected. Diurnal variation in $\dot{\text{V}}{{\text{O}}_2}$ kinetics has been previously observed during moderate-intensity cycling [61], but not during high-intensity running [62]. There is evidence that decreased HR_max_ due to intensified training is accompanied by a reduction in ${\dot{\text{V}}}{\text{O}}_{2\max}$ [53, 63], which could also have affected time in ${\text{Z}}{3_{\dot{\text{V}}\text{O}_2}}$. As of yet, there is insufficient research on double HIIT sessions as part of a HIIT-SM in general, and their effect on HR, $\dot{\text{V}}{{\text{O}}_2}$, and their interaction.

### Correlation Analysis

It is believed that a dose–response relationship exists between the total time spend in Z3, i.e., at or close to ${\dot{\text{V}}}{\text{O}}_{2\max}$, and improvements in ${\dot{\text{V}}}{\text{O}}_{2\max}$ [33, 36, 64]. Interestingly, the current study found no relationships between the time spent in Z3_HR_, ${\text{Z}}{3_{\dot{\text{V}}\text{O}_2}}$, Z3_P_ and changes in performance parameters. Comparisons with previous research are limited as studies in this field mostly focused on interventions with longer periods of time (4–8 weeks), where athletes normally perform 2–3 HIIT sessions per week. To the best of our knowledge, there is only one study investigating a dose–response relationship during a HIIT-SM. Rønnestad et al. [30] performed five HIIT (6 × 5 min) sessions within six days with elite cross-country skiers who accumulated on average between ~ 12 to  ~ 16 min ≥ 90% ${\dot{\text{V}}}{\text{O}}_{2\max}$ and ~ 18 to  ~ 23 min ≥ 90% HR_max_ in sessions one, two, and five. Correlation analysis revealed a tendency that time ≥ 90% ${\dot{\text{V}}}{\text{O}}_{2\max}$ could estimate the improvement in ${\dot{\text{V}}}{\text{O}}_{2\max}$ (r = 0.54, p = 0.071), while it could not explain changes in other performance parameters, e.g., velocity at 4 mmol L^−1^ La (r = 0.211, p = 0.511). Our results, with athletes spending on average ~ 11 to  ~ 13 min in ${\text{Z}}{3_{\dot{\text{V}}\text{O}_2}}$ during sessions two, three, nine, and ten do not support a clear relationship between time in ${\text{Z}}{3_{\dot{\text{V}}\text{O}_2}}$ and Δ ${\dot{\text{V}}}{\text{O}}_{2\max}$ (r_s_ = 0.24, p = 0.335). It needs to be mentioned, that the comparison between these two studies should be treated with caution as we opted to utilize $\dot{\text{V}}{{\text{O}}_2}$ at 90% HR_max_ instead of 90% ${\dot{\text{V}}}{\text{O}}_{2\max}$ as a threshold for ${\text{Z}}{3_{\dot{\text{V}}\text{O}_2}}$. This was done to comply with the interval intensity prescriptions and to standardize zone determination across all intensity measures.

Previous studies have primarily investigated the dose–response relationship between internal measures (HR, $\dot{\text{V}}{{\text{O}}_2}$) and the development of ${\dot{\text{V}}}{\text{O}}_{2\max}$ as a surrogate of actual endurance performance [32, 36], which we cannot confirm with our data. However, there were moderate correlations between time in Z3_V_ and Δ PPO (r_s_ = 0.52, p = 0.028) as well as Δ TT (r_s_ = -0.47, p = 0.049), estimating that athletes who spent more time in Z3_V_ during the HIIT-SM were more likely to improve their PPO and TT_5km_ performance. Therefore, these results illustrate the relationship between an external measure (velocity) and the actual endurance performance, i.e., TT_5km_. This is another example why measuring intensity based on velocity might be more appropriate compared to HR in a running based HIIT-SM setting. Velocity represents the actual work performed more reliably, at least on flat terrain, while HR tends to underestimate the intensity and load; a discrepancy that becomes even more evident with increased parasympathicotonia and accumulating fatigue (Fig. 4A). These are important implications for athletes and coaches, suggesting that the effectiveness of HIIT-SM should not be evaluated on the basis of time in Z3_HR_. Since we decided to measure $\dot{\text{V}}{{\text{O}}_2}$ in only 4 out of 10 sessions, it remains to be investigated whether time in ${\text{Z}}{3_{\dot{\text{V}}\text{O}_2}}$ accumulated over all HIIT sessions is a better predictor of change in endurance-related parameters, which should be the subject of future studies.

${\dot{\text{V}}}{\text{O}}_{2\max}$, PPO, and LT measured at baseline turned out to be strong predictors of TT1 performance being in line with previous literature [65, 66]. Although, there is robust evidence that running economy is important for running performance [45], we only found a moderate relationship with TT1 (r_s_ = 0.41, p = 0.094), which aligns with previously results for the same TT distance (r = 0.44 [67], r = 0.39 [68]). Interestingly, baseline running economy turned out to be a good estimate for Δ TT (r_s_ = − 0.68, p = 0.003). This indicates that less efficient participants were more likely to improve their TT_5km_ time through HIIT-SM than those who already had a better running economy. This was also reflected in the relationship between baseline and Δ running economy (r_s_ = − 0.69, p = 0.002). These relationships illustrate the potential for improving key performance parameters using HIIT-SM for endurance-trained athletes. However, it appears that comparatively less trained athletes from our cohort benefit to a greater extent.

It is hypothesized, that cardiac chamber sizes serve as a predictor for a high ${\dot{\text{V}}}{\text{O}}_{2\max}$, suggesting that individuals with smaller cardiac chambers may exhibit greater potential for performance improvement following a HIIT intervention. Several studies have indicated significant, weak to strong correlations, mainly between baseline resting LV EDV or LV mass and ${\dot{\text{V}}}{\text{O}}_{2\max}$ [69, 70]. However, we did not find significant correlations between resting cardiac chamber size measurements and baseline endurance performance variables. This might be attributed to the relatively homogenous study population and the small sample size. Another reason for the weak correlation between cardiac chamber sizes and endurance performance could also be that myocardial compliance plays a major role in stroke volume generation and subsequently ${\dot{\text{V}}}{\text{O}}_{2\max}$ [71], indicating that chamber sizes at rest are actually less important compared to how they enlarge during exercise.

Furthermore, our analysis revealed no significant correlations between cardiac geometry variables and change scores with the exception of LA ESV and Δ TT, where a negative correlation was observed (2D: r_s_ = − 0.48, p = 0.045, 3D: r_s_ = − 0.46, p = 0.066). This suggests that participants with smaller LA were more likely to improve their TT time through HIIT-SM than those with larger LA. Several studies have indicated the importance of atrial contribution to stroke volume generation but also atrial wall stress during prolonged and intensive endurance exercise bouts [72, 73]. This underscores the potential value of monitoring LA size for guiding exercise recommendations. However, further research with larger sample size and measurements during exercise is needed to comprehensively explore the role of cardiac geometry, particularly size of the LA, which has the potential to improve personalized training recommendations.

### Limitations

Although, to date, there seems to be no evidence that biological sex affects TID, the uneven distribution of female participants between the groups needs to be considered. This disparity is mainly attributed to dropouts and data quality checks, which unfortunately reduced the number of female athletes. As the CG was instructed to continue with their regular training, including other sports beside running, comparisons based on velocity and power with the intervention groups were not feasible, potentially missing out on valuable additional information. When interpreting the results of the correlation analysis, it is important to consider that intervention groups were pooled to increase sample size, knowing that one half has performed more LIT than the other half potentially impacting the outcomes. However, to give the reader a more complete picture, we have added a separate analysis for each group in Supplement 3. Finally, there was no detailed monitoring of subjective perception in the form of RPE during the HIIT-SM, potentially missing out on valuable data.

## Conclusion

We conclude that the selection of intensity measure strongly affects the quantification of TID for HIIT-SM in endurance-trained athletes. Informed decisions based on TID_HR_ should be treated with caution as HR underestimates the actual training intensity during a HIIT-SM. Time in Z3_V_ appeared to be valuable in detecting dose–response relationships, indicating that athletes who spent more time in the “red zone” determined by running velocity may achieve higher improvements in PPO and TT performance. We recommend contextualizing a combination of external and internal measures to evaluate the training data of HIIT-SM’s. Cardiac geometry turned out to be insufficient in estimating changes in endurance performance parameters induced by HIIT-SM, except for the relationship between LA ESV and changes in TT performance, which should be investigated in future studies with different designs and populations. 

## Supplementary Information

Additional file 1

Additional file 2

Additional file 3

## Data Availability

The datasets used and/or analysed during the current study are available from the corresponding author on reasonable request.

## Abbreviations

TID: Training intensity distribution
Z: Zone
LIT: Low-intensity training
HR: Heart rate
$${\dot{\text{V}}}{\textrm{O}}_{2}$$: Oxygen uptake
La: Blood lactate
RPE: Rating of perceived exertion
${\dot{\text{V}}}{\text{O}}_{2\max}$: Maximal oxygen uptake
HIIT: High-intensity interval training
HIIT-SM: High-intensity interval training shock microcycle
HR_max_: Maximal heart rate
LV: Left ventricle
LA: Left atrial
TID_V_: TID based on running velocity
TID_P_: TID based on running power
TID_HR_: TID based on heart rate
Z3_V_: Time in zone 3 based on velocity
Z3_P_: Time in zone 3 based on running power
Z3_HR_: Time in zone 3 based on heart rate
${\text{Z3}}_{\dot{\text{VO}}_2}$: Time in zone 3 based on oxygen uptake
TT_5km_: 5-km time trial
TB: Best out of three post measurements
EDD: End-diastolic diameter
EDV: End-diastolic volume
ESV: End-systolic volume
RV: Right ventricular
RA: Right atrial
PPO: Peak power output
LT: Lactate threshold
SD: Standard deviation
SEM: Standard error of the mean
_p_η^2^: Partial eta squared
Δ: Change score
