# Supplementary material for: Training Intensity Distribution of a 7-Day HIIT Shock Microcycle: Is Time in the “Red Zone” Crucial for Maximizing Endurance Performance? A Randomized Controlled Trial
Source: Sports Med Open. 2024 Sep 5;10:97. doi: 10.1186/s40798-024-00761-1 (PMC11377407; doi:10.1186/s40798-024-00761-1)
Supplement: Supplementary file 1 — Additional file 1 [file 40798_2024_761_MOESM1_ESM.docx]

**Supplementary Information**

**Study title:**

“Training Intensity Distribution of a 7-day HIIT Shock Microcycle: Is time in the “red zone” crucial for maximizing endurance performance? A Randomized Controlled Trial”

**Authors:**

Tilmann Strepp^1^*, Julia C. Blumkaitis^1^, Mahdi Sareban^2^, Thomas Leonhard Stöggl^1,3^, Nils Haller^1,4^

^1^ Department of Sport and Exercise Science, University of Salzburg, Salzburg, Austria

^2^ University Institute of Sports Medicine, Prevention and Rehabilitation, Paracelsus Medical University, Salzburg, Austria.

^3^ Red Bull Athlete Performance Center, Thalgau, Austria

^4^ Department of Sport Medicine, Rehabilitation and Disease Prevention, Johannes Gutenberg University of Mainz, Mainz, Germany

**For publication in:**

Sports Medicine - Open in “Original Research Articles”

**Corresponding Author:**

Tilmann Strepp

Email: tilmann.strepp@plus.ac.at

**Supplement 1:**

**Eligibility Criteria**

Inclusion Criteria:

- Female or male
- aged 18-45 years
- Proof of physical fitness (e.g. sports medical examination required) for measurements with higher intensities (e.g. endurance tests, competition simulation)
- Competition experience at the national or international level in an endurance sport
- VO2max ≥50ml/kg/min for females; ≥55 ml/kg/min for males or a 5-kilometer (km) time trial performance of ≤ 20:00 min (female), or ≤ 18:30 min (male)

Exclusion Criteria:

- Systemic disease or other known pathology in the organs: heart, lungs, kidney, stomach, spleen, liver, gall bladder, and intestines.
- Evidence of pulmonary disease: forced expiratory volume in one second/forced expiratory volume < 70% with/without symptoms (cough, sputum) or other evidence of pulmonological disease.
- Diabetes II.
- Neurological or psychological disease of any kind.
- Currently undergoing medical or psycho-therapeutic treatment.
- Health condition that does not allow regular participation in the training forms (e.g. acute illnesses such as fever or other flu-like infections within the last 7 days before the start of the study), orthopedic diseases, injuries to the muscular, bone, joint or tendon apparatus within the last three months.
- Alcohol or drug abuse.
- Already high training volume with high intensity training (more than 2 weekly training sessions of high-intensity training)
