# Supplementary material for: Training Intensity Distribution of a 7-Day HIIT Shock Microcycle: Is Time in the “Red Zone” Crucial for Maximizing Endurance Performance? A Randomized Controlled Trial
Source: Sports Med Open. 2024 Sep 5;10:97. doi: 10.1186/s40798-024-00761-1 (PMC11377407; doi:10.1186/s40798-024-00761-1)
Supplement: Supplementary file 2 — Additional file 2 [file 40798_2024_761_MOESM2_ESM.docx]

**Supplementary Information**

**Study title:**

“Training Intensity Distribution of a 7-day HIIT Shock Microcycle: Is time in the “red zone” crucial for maximizing endurance performance? A Randomized Controlled Trial”

**Authors:**

Tilmann Strepp^1^*, Julia C. Blumkaitis^1^, Mahdi Sareban^2^, Thomas Leonhard Stöggl^1,3^, Nils Haller^1,4^

^1^ Department of Sport and Exercise Science, University of Salzburg, Salzburg, Austria

^2^ University Institute of Sports Medicine, Prevention and Rehabilitation, Paracelsus Medical University, Salzburg, Austria.

^3^ Red Bull Athlete Performance Center, Thalgau, Austria

^4^ Department of Sport Medicine, Rehabilitation and Disease Prevention, Johannes Gutenberg University of Mainz, Mainz, Germany

**For publication in:**

Sports Medicine - Open in “Original Research Articles”

**Corresponding Author:**

Tilmann Strepp

Email: tilmann.strepp@plus.ac.at

**Supplement 2:**

**
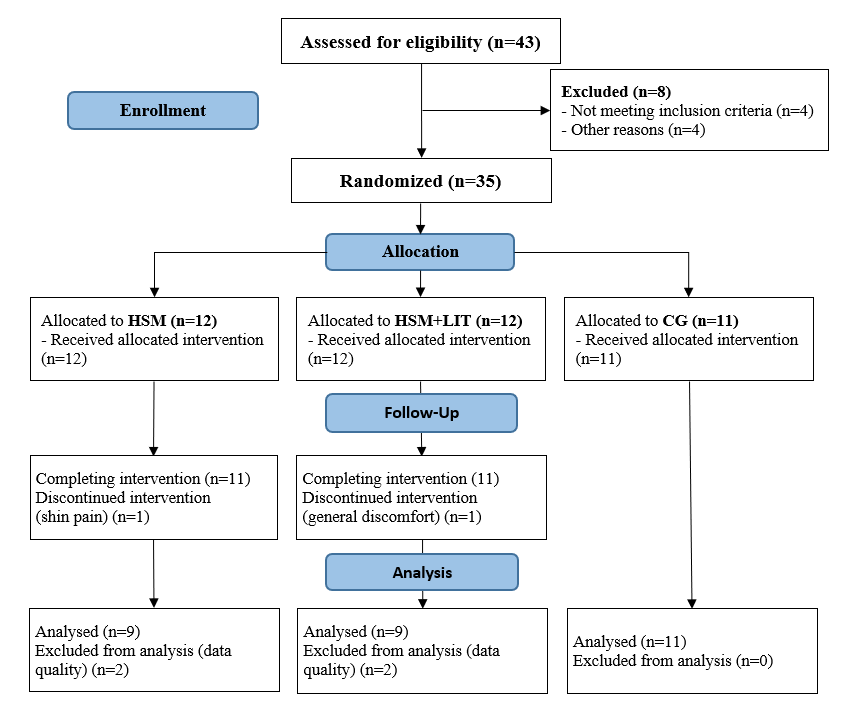
**

Participant flow-chart. HSM, high-intensity shock microcycle; HSM+LIT, high-intensity shock microcycle with additional low-intensity training; CG, control group
