# Supplementary material for: Training Intensity Distribution of a 7-Day HIIT Shock Microcycle: Is Time in the “Red Zone” Crucial for Maximizing Endurance Performance? A Randomized Controlled Trial
Source: Sports Med Open. 2024 Sep 5;10:97. doi: 10.1186/s40798-024-00761-1 (PMC11377407; doi:10.1186/s40798-024-00761-1)
Supplement: Supplementary file 3 — Additional file 3 [file 40798_2024_761_MOESM3_ESM.docx]

**Supplementary Information**

**Study title:**

“Training Intensity Distribution of a 7-day HIIT Shock Microcycle: Is time in the “red zone” crucial for maximizing endurance performance? A Randomized Controlled Trial”

**Authors:**

Tilmann Strepp^1^*, Julia C. Blumkaitis^1^, Mahdi Sareban^2^, Thomas Leonhard Stöggl^1,3^, Nils Haller^1,4^

^1^ Department of Sport and Exercise Science, University of Salzburg, Salzburg, Austria

^2^ University Institute of Sports Medicine, Prevention and Rehabilitation, Paracelsus Medical University, Salzburg, Austria.

^3^ Red Bull Athlete Performance Center, Thalgau, Austria

^4^ Department of Sport Medicine, Rehabilitation and Disease Prevention, Johannes Gutenberg University of Mainz, Mainz, Germany

**For publication in:**

Sports Medicine - Open in “Original Research Articles”

**Corresponding Author:**

Tilmann Strepp

Email: tilmann.strepp@plus.ac.at

**Supplement 3:**

**
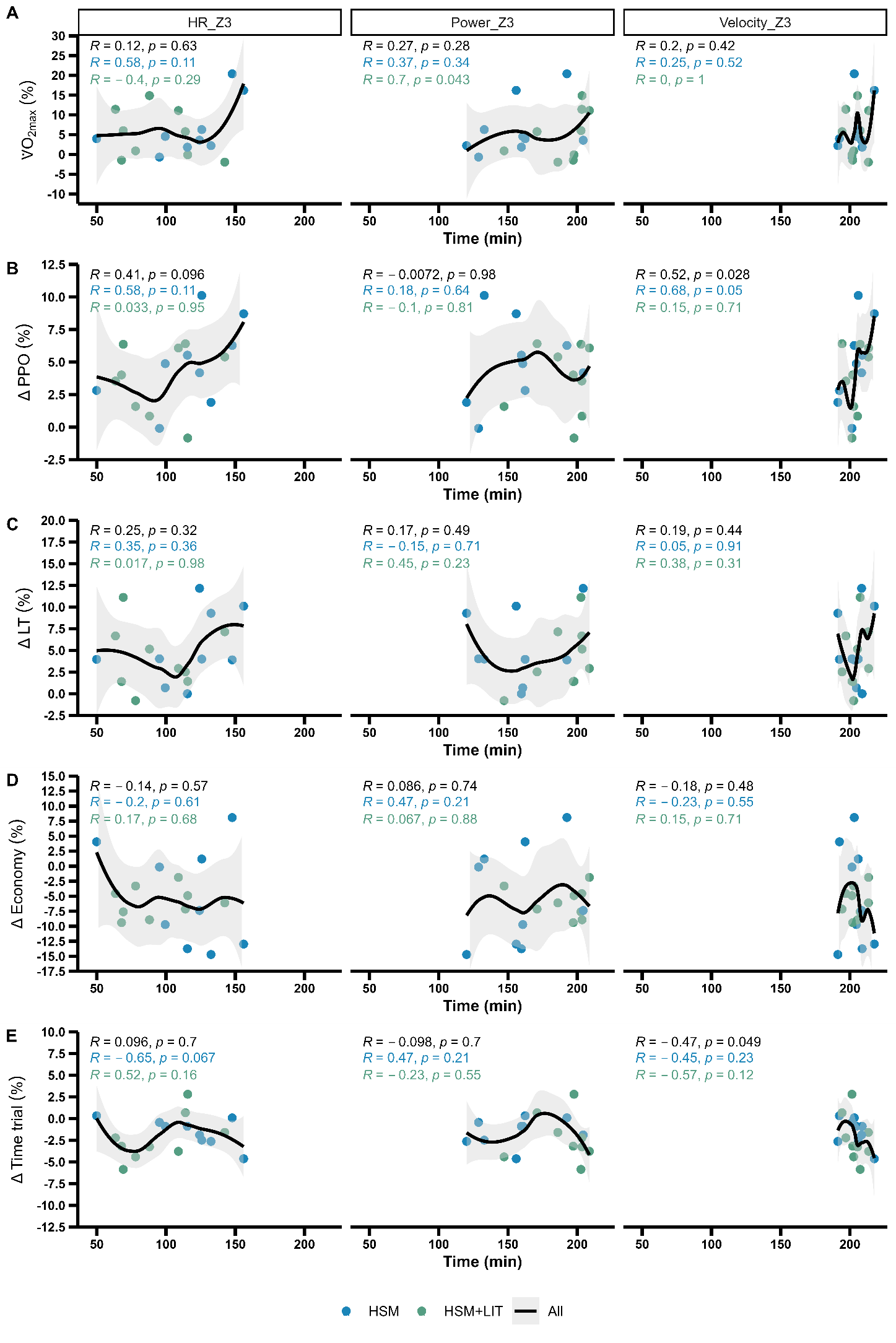
**

Spearman rank correlations between changes in endurance performance parameters and time in zone 3 (Z3) measured by heart rate (HR), running power, and running velocity for each intervention group as well as pooled. Black lines, “loess” function i.e., locally estimated scatterplot smoothing for the pooled sample; Grey band, 95% confidence interval; HSM, high-intensity shock microcycle; HSM+LIT, high-intensity shock microcycle with additional low-intensity training; V̇O_2max_, maximal oxygen uptake; Δ, changes from pre to best posttest; PPO, peak power output; LT, lactate threshold.


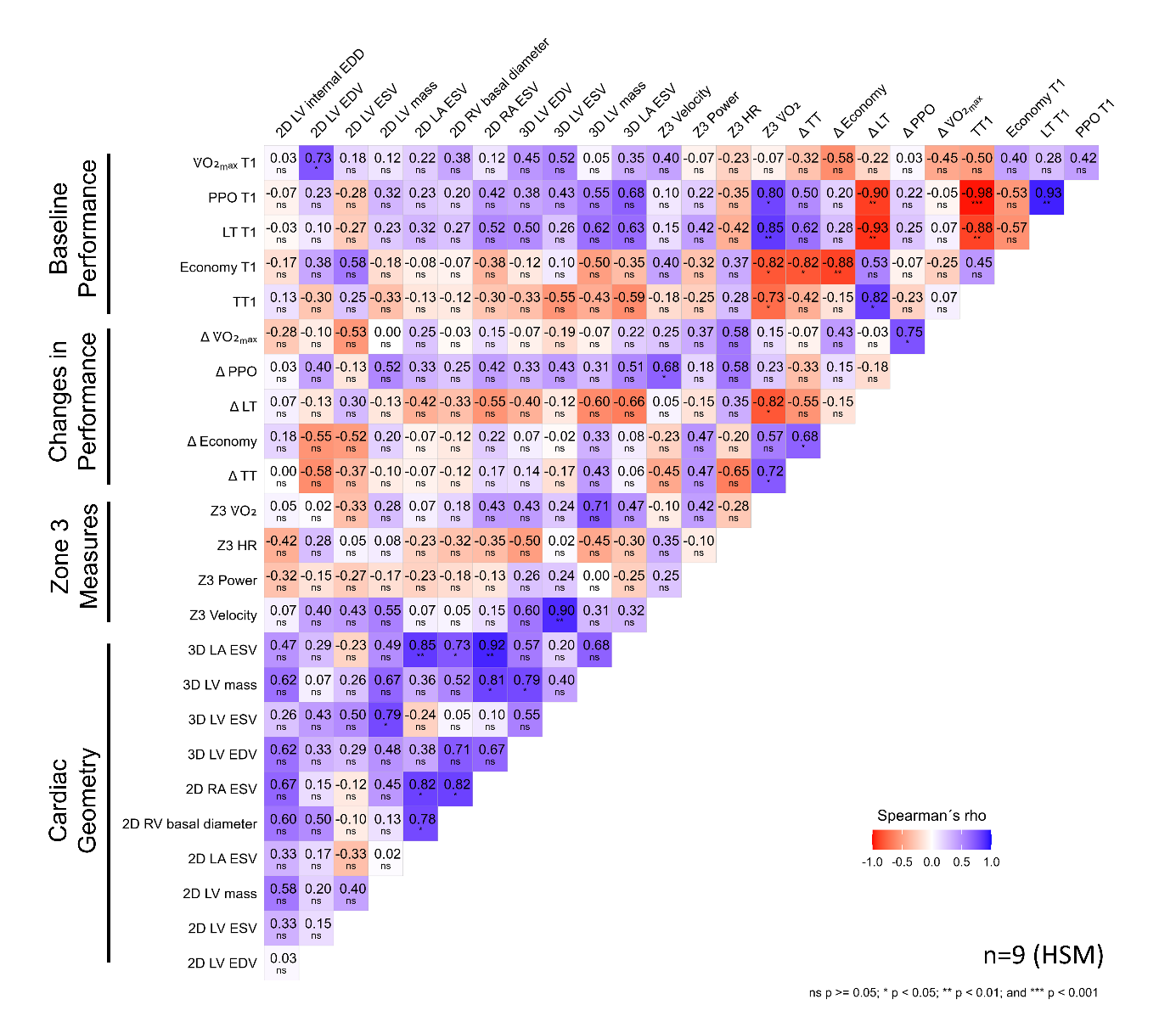


Correlation matrix (spearman rank) with baseline values and changes in performance measures, total time in zone 3 in minutes (Z3) determined with different intensity measures, and cardiac geometry variables for high-intensity interval training shock microcycle group (HSM). T1, initial cardiopulmonary exercise testing; V̇O_2max_, maximal oxygen uptake; Δ, changes from pre to best posttest; PPO, peak power output; LT, lactate threshold; TT, 5km time trial; 2D, two-dimensional; 3D, three-dimensional; LV internal EDD, left ventricular internal end-diastolic diameter; LV EDV, left ventricular end-diastolic volume; LV ESV, left ventricular end-systolic volume; LV mass, left ventricular mass; LA ESV, left atrial end-systolic volume; RV, right ventricular; RA ESV, right atrial end-systolic volume. All cardiac measurements were indexed to body surface area.

**
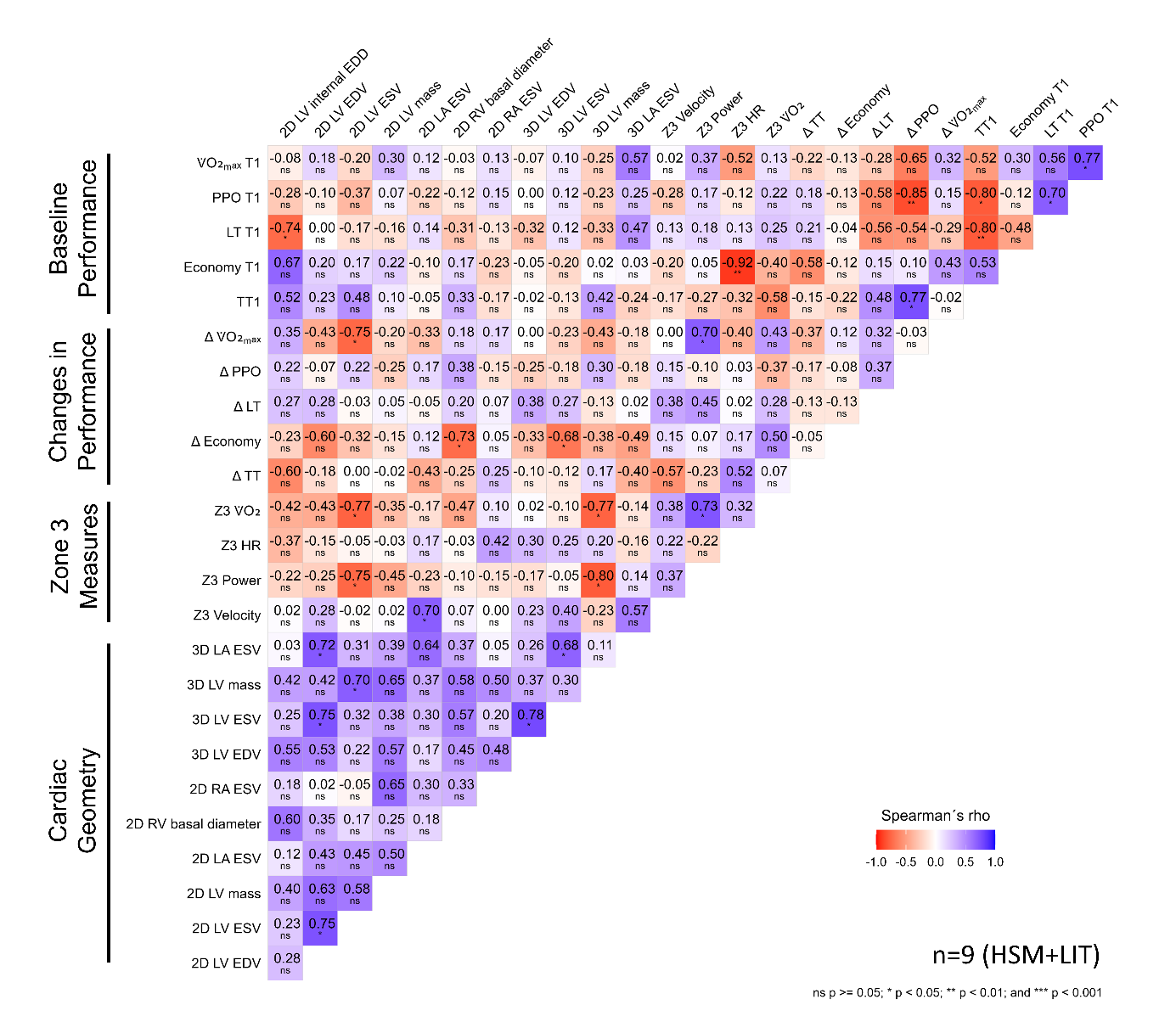
**

Correlation matrix (spearman rank) with baseline values and changes in performance measures, total time in zone 3 in minutes (Z3) determined with different intensity measures, and cardiac geometry variables for high-intensity interval training shock microcycle group with additional low-intensity training (HSM+LIT). T1, initial cardiopulmonary exercise testing; V̇O_2max_, maximal oxygen uptake; Δ, changes from pre to best posttest; PPO, peak power output; LT, lactate threshold; TT, 5km time trial; 2D, two-dimensional; 3D, three-dimensional; LV internal EDD, left ventricular internal end-diastolic diameter; LV EDV, left ventricular end-diastolic volume; LV ESV, left ventricular end-systolic volume; LV mass, left ventricular mass; LA ESV, left atrial end-systolic volume; RV, right ventricular; RA ESV, right atrial end-systolic volume. All cardiac measurements were indexed to body surface area.
